# Letter Concerning Dallas Meeting of Southwestern Medical Association

**Published:** 1918-08

**Authors:** M. M. Smith

**Affiliations:** Chairman Arrangements Committee; Dallas, Texas


**Letter About Dallas Meeting Southwest Medical Association.**

Dallas, Texas, July 12, 1918.

Dr. Fred H. Clark, Secretary,
The Medical Association of the Southwest,
El Reno, Oklahoma.

Dear Dr. Clark: As chairman of the Arrangements Committee for the next meeting of the Medical Association of the Southwest, I am writing to say that the medical profession of Dallas has already begun actively to arrange for the meeting of this association scheduled to be held October 15, 16 and 17, 1918, in this city.

An exceptionally strong committee has been appointed whose duty it will be to solicit new members for the association and to urge the attendance of present members upon the Dallas meeting. It is the determination of the profession of Dallas that the promise I made at the meeting of the Medical Association of the Southwest in Ft. Smith, Ark., and which I repeated at the Kansas City meeting of the association last year, shall be kept—namely, that more new members will be added to the association when it is held in Texas, than were obtained from both the other states of Arkansas and Missouri in the previous years.

I am sure that a committee has been selected that will see to it, that this promise is fulfilled in every particular.

I desire to urge upon you, through the journal, Dr. Clark, that all interested members of the association from Missouri, Kansas, Oklahoma and Arkansas, arrange to form state organizations among themselves and select regular chairmen and secretaries, for the purpose of urging as many members from their respective states as possible to plan to attend the Dallas convention, for it is our purpose to make this the largest attended and the best, if possible, meeting of the association in its entire history, notwithstanding the war.

It is further our purpose to furnish a program that will be educational and instructive and of such great value, that the profession cannot well afford to miss it and in addition to this, it is our desire to prepare and furnish entertainment for the visiting ladies and other guests, that will be appropriate and suitable in order to make this a most enjoyable occasion.

I desire to urge upon you, Dr. Clark, and through you, the profession all over the territory represented in this association, that they all join hands with the Dallas and Texas profession, in making this 1918 meeting a wonderful success.

I shall write you soon again with reference to further progress, and assuring you of my hearty co-operation in the work, remain,

Fraternally yours,

M. M. Smith,
Chairman Arrangements Committee.

